# Gut Microbiota Composition and Predicted Functional Profiles: Functional Adaptation of Overwintering Whooper Swans (*Cygnus cygnus*) Across Habitats with Contrasting Anthropogenic Disturbance

**DOI:** 10.3390/ani16172638

**Published:** 2026-08-23

**Authors:** Liangliang Yang, Qiyu Zeng, Yijie Liu, Yongjun Zhang, Qing L. Cao

**Affiliations:** 1 State Key Laboratory of Wetland Conservation and Restoration, Ecology and Nature Conservation Institute, Chinese Academy of Forestry, Beijing 100091, China; kddliang1982@126.com; 2 Xinjiang Laboratory of Special Species Conservation and Regulatory Biology, School of Life-Science, Xinjiang Normal University, Urumqi 830017, China; 17711344912@163.com (Q.Z.); 15929384032@163.com (Y.L.); 3 Department of Ecology and Evolutionary Biology, Princeton University, Princeton, NJ 08544, USA

**Keywords:** whooper swan, gut microbiota, urban wetland, riverine wetland, 16S rRNA sequencing, wildlife conservation

## Abstract

Gut microbiota plays an important role in host metabolism, immunity, and environmental adaptation, yet its response to contrasting wintering habitats in migratory waterbirds remains poorly understood. We compared the gut microbiota of whooper swans (Cygnus cygnus) wintering in a natural riverine habitat (Swan Bay, Hejing County) and an urban wetland habitat (Worker’s Park, Korla City) in Xinjiang, China. A total of 34 fresh fecal samples were analyzed using 16S rRNA gene sequencing. We identified 2207 operational taxonomic units (OTUs), of which 734 were shared between habitats, indicating the presence of a conserved core microbiota. The urban wetland group exhibited a significantly higher Simpson index, whereas beta diversity analysis revealed significant differences in community structure between habitats (*p* < 0.05). Both groups were dominated by Firmicutes, *Fusobacteriota*, Proteobacteria, Campylobacterota, and Bacteroidota, although their relative abundances differed markedly. LEfSe analysis identified distinct habitat-associated taxa, and PICRUSt2 prediction suggested greater enrichment of metabolism-related pathways in the natural riverine group. Overall, wintering habitat was associated with differences in gut microbial composition and predicted function, providing new insight into the ecological adaptation of migratory waterbirds under contrasting environmental conditions.

## Simple Summary

Whooper swans (*Cygnus cygnus*) are migratory waterbirds that spend the winter in habitats ranging from natural rivers to urban wetlands. As human activities increasingly alter wetland environments, understanding how these changes affect wildlife has become an important conservation issue. Microorganisms living in the digestive tract play important roles in nutrition, health, and adaptation to environmental conditions. In this study, we compared the gut microbial communities of whooper swans wintering in a natural river habitat and an urban wetland in Xinjiang, China. We found that swans from the two habitats shared many common microorganisms, but differed in microbial diversity, community composition, and predicted biological functions. Swans wintering in the natural river habitat harbored more microorganisms associated with nutrient metabolism, whereas those living in the urban wetland showed a greater abundance of microorganisms commonly linked to environmental exposure. These findings suggest that habitat conditions are strongly associated with variations in the intestinal microbiota of whooper swans, which may correlate with their physiological adjustments across diverse wintering scenarios. Our work offers preliminary insights relevant to wetland preservation, wildlife health monitoring, and the adaptive management of wintering swan populations in anthropogenic landscapes.

## 1. Introduction

Gut microbiota constitutes a crucial component of the host-associated microecosystem and plays essential roles in nutrient metabolism, energy acquisition, immune regulation, and maintenance of physiological homeostasis [1,2,3]. Increasing evidence has demonstrated that gut microbial communities are shaped not only by host phylogeny and physiological status, but also by environmental conditions, dietary resources, and anthropogenic disturbances [4,5,6,7]. In migratory birds, seasonal movements across geographically distinct habitats expose individuals to substantial environmental heterogeneity, potentially leading to shifts in gut microbial composition and function. Such microbial changes may further influence host nutritional adaptation, ecological fitness, and pathogen transmission risk [8,9,10]. Consequently, understanding the ecological drivers of gut microbiota variation in migratory waterbirds has become an important topic in avian ecology and wildlife conservation research.

The whooper swan (*Cygnus cygnus*) is a large migratory waterbird belonging to the family Anatidae and is listed as a nationally protected species in China [11]. As a typical wintering migrant, whooper swans annually migrate along fixed flyways and overwinter in wetlands distributed across different ecological regions. Previous studies on this species have mainly focused on migration ecology, population dynamics, habitat utilization, dietary composition, and conservation management [12,13,14]. However, the overwintering period is a critical stage in the annual life cycle, during which individuals must maintain physiological homeostasis under low temperature conditions while accumulating sufficient energy reserves for subsequent migration and reproduction [15,16,17]. Therefore, habitat quality during overwintering is closely linked to individual fitness and population persistence.

Although wildlife microbiome research has expanded rapidly in recent years, studies investigating gut microbial variation in whooper swans under different overwintering environments remain limited [18,19]. More importantly, it remains unclear whether urbanized overwintering habitats alter gut microbial community structure and metabolic functional potential in migratory waterbirds. Urban wetlands are increasingly serving as alternative wintering sites; however, these environments differ fundamentally from natural wetlands. Ecologically, urbanization alters wetland hydrological properties, shifts natural vegetation toward artificial food supplementation, and increases exposure to human-associated microbes and pollutants. These environmental alterations can directly modify host foraging behavior, physiological stress, and dietary intake. Because the gut microbiota responds dynamically to environmental inputs, such habitat-driven shifts are expected to alter host–microbe interactions. In this context, “environmentally associated microorganisms” refer to taxa transiently or persistently acquired through direct contact with external environmental matrices (e.g., ingestion of water, sediment, or artificial food items) rather than host-adapted core symbionts. Compared with natural riverine wetlands, urban habitats may therefore exert distinct selective pressures on host-associated microbial communities. Nevertheless, direct comparative evidence between natural and urban overwintering habitats in migratory swans is still lacking.

Xinjiang, northwestern China, provides an ideal natural framework for addressing this question because both natural riverine overwintering habitats and urban artificial wetlands coexist within the region. Swan Bay in Hejing County is located along the natural section of the Kaidu River and is characterized by relatively intact wetland ecosystems with comparatively low human disturbance. In contrast, Workers’ Park in Korla City represents a typical urban artificial wetland embedded within a densely populated urban landscape and subjected to long-term human management and recreational activities. Despite substantial differences in landscape structure, environmental conditions, and anthropogenic disturbance intensity, both habitats support relatively stable overwintering populations of whooper swans. Therefore, the present study aimed to compare the gut microbial community composition, diversity patterns, and predicted functional profiles of overwintering whooper swans inhabiting natural riverine and urban wetland environments using 16S rRNA gene high-throughput sequencing. Specifically, this study addressed the following questions: (1) whether different overwintering habitats significantly influence gut microbial diversity and community structure in whooper swans; (2) whether urban environments promote the enrichment of environmentally associated or potentially pathogenic microorganisms; and (3) whether habitat differences are associated with shifts in microbial metabolic functional potential.

We hypothesized that urban overwintering habitats—associated with altered food availability, water conditions, and anthropogenic contact—would correlate with shifts in host physiological exposures and a restructuring of the gut microbiota in whooper swans. These habitat differences were expected to correspond to increased microbial diversity (potentially driven by elevated environmental inputs), shifted community composition, and a higher representation of environmentally associated or opportunistic pathogenic taxa compared to natural riverine habitats. In contrast, swans overwintering in natural habitats were expected to maintain microbial profiles more closely aligned with nutrient metabolism and natural dietary foraging. The findings of this study provide preliminary microbiological insights into wild waterbird ecology, supporting overwintering habitat conservation, urban wetland management, and wildlife health monitoring in Xinjiang.

## 2. Materials and Methods

### 2.1. Study Species and Study Sites

**Figure 1:**
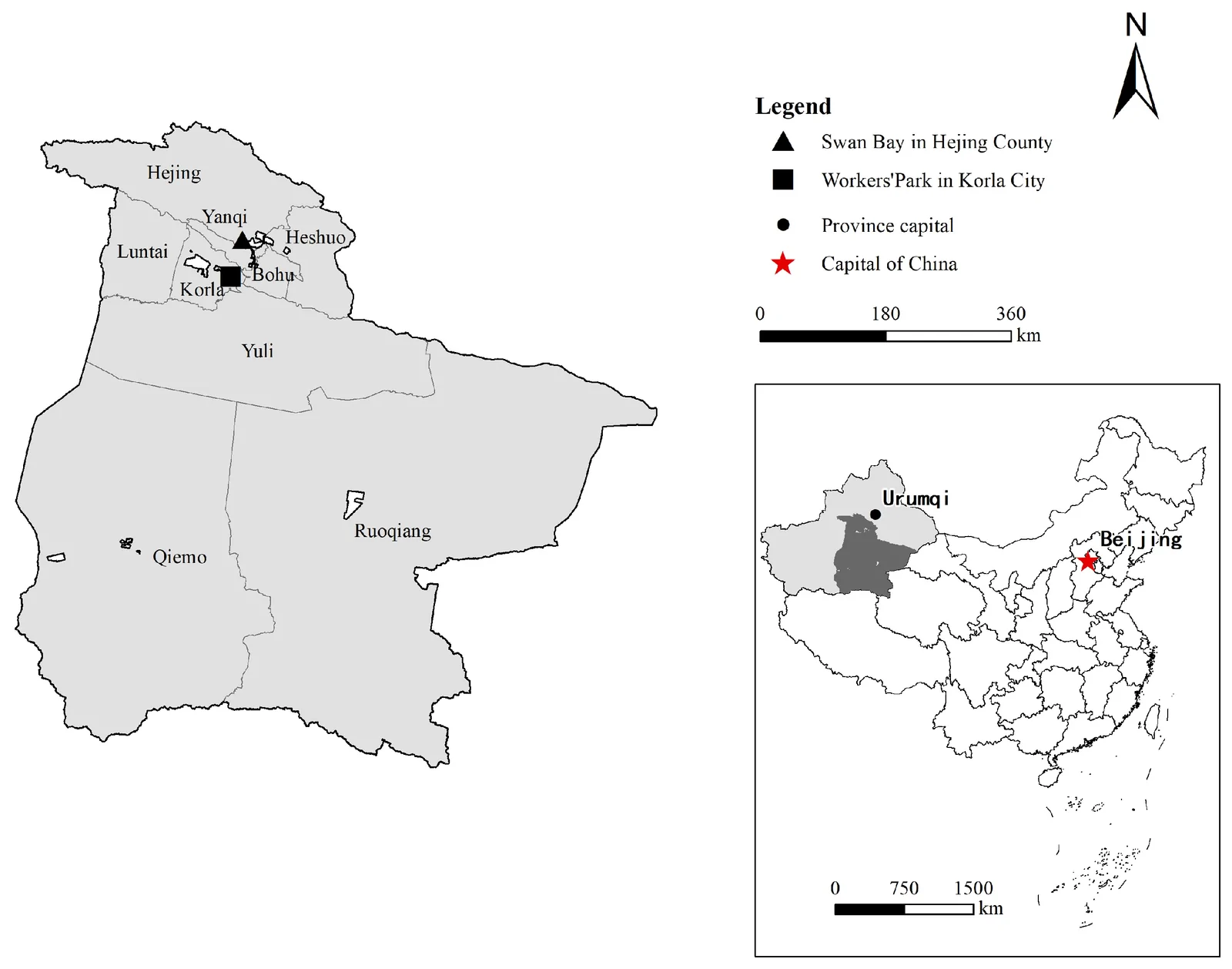
Geographical location of whooper swan study sites.

This study focused on overwintering whooper swans (*Cygnus cygnus*) inhabiting Hejing County and Korla City in the Bayingolin Mongolian Autonomous Prefecture, Xinjiang, China (Figure 1). Both sites represent important overwintering habitats for whooper swans in northwestern China, supporting stable annual populations during the wintering period. In this region, whooper swans typically arrive at both overwintering sites in late October to early November and depart by mid-March. To minimize transient physiological fluctuations associated with recent migration and dietary transitions during early arrival, the current comparative analysis specifically focuses on the peak overwintering period (December), when swan populations have fully acclimated to local habitats and individual movements are highly stable. Due to the geographic distance (~60 km) and strong wintering site fidelity, the two overwintering sub-populations remain relatively isolated with minimal population mixing during the sampling window. Although both habitats support overwintering swans, they differ substantially in environmental characteristics, dietary resource regimes, and levels of anthropogenic disturbance.

**Figure 2:**
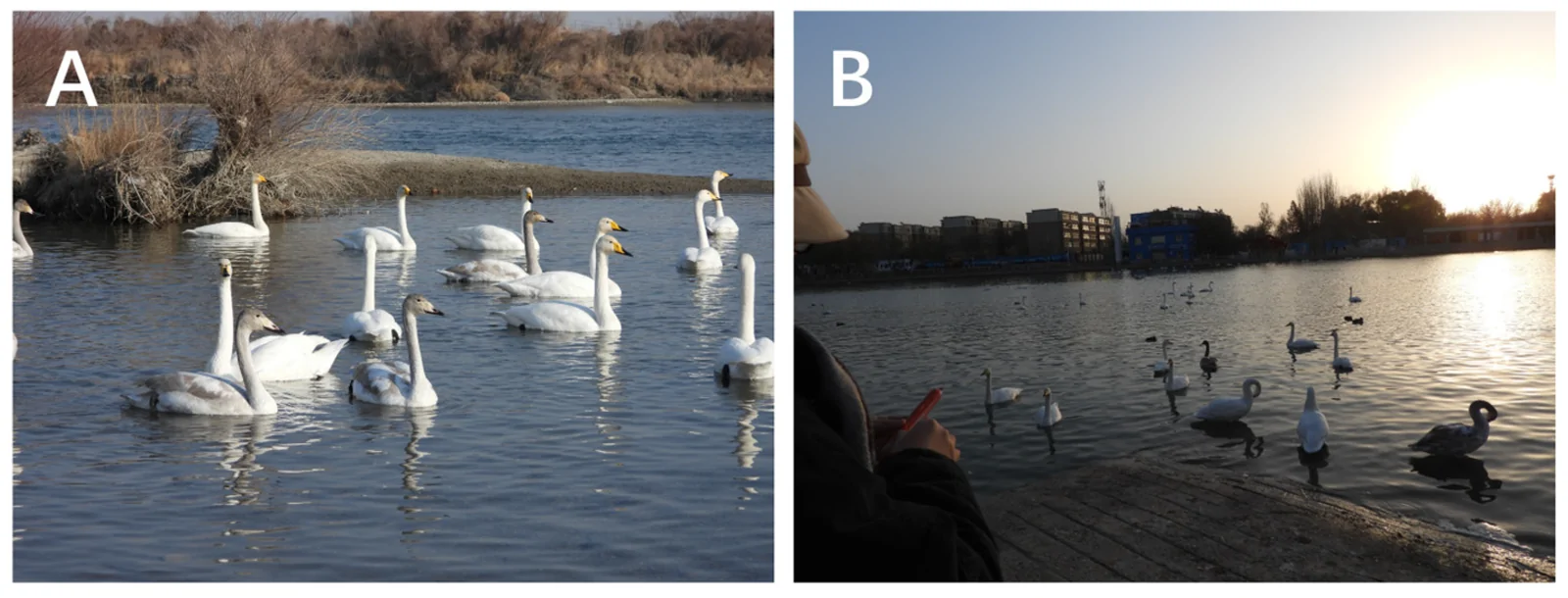
Representative field habitats of overwintering whooper swans (*Cygnus cygnus*) at the two sampling sites. (**A**) Natural riverine wetland in Swan Bay, Hejing County (SBHC), featuring natural river channels, unfragmented riparian vegetation, and minimal anthropogenic disturbance. (**B**) Urban artificial park wetland in Workers’ Park, Korla City (WPKC), characterized by concrete banks and shorelines, surrounding urban infrastructure, close-range human interaction, and regular artificial food supplementation.

Swan Bay in Hejing County (SBHC; 42°14′ N, 86°24′ E) is located along the natural section of the Kaidu River and represents a typical natural riverine wetland habitat with relatively intact ecological conditions (Figure 2A). The area is dominated by natural river channels and continuous riparian wetlands, where hydrological patterns, shoreline structures, and surrounding landscape configurations largely retain their original habitat characteristics. The aquatic vegetation is composed primarily of native submerged and emergent macrophytes (e.g., *Salix* spp. and *Phragmites australis*), providing natural foraging grounds. Human disturbance in this site is minimal, largely restricted to regulated eco-tourism and birdwatching activities along designated buffer zones. During winter, stable open-water surfaces, continuous riparian zones, and minimal human interference provide suitable conditions for resting, foraging, and roosting of whooper swans. Currently, this area supports a relatively stable overwintering population of approximately 400 individuals annually and has gradually developed into an ecotourism destination integrating wildlife conservation and birdwatching activities.

In contrast, Workers’ Park in Korla City (WPKC; 41°43′ N, 86°10′ E) represents a typical urban artificial park wetland (covering an open-water area of approximately 0.18 km^2^, Figure 2B) embedded within a densely populated urban built-up landscape. The site is surrounded by high-density commercial and residential zones, experiencing intense anthropogenic disturbance and regular recreational visitation, with daily peak human activity concentrated between 11:00 and 18:00 (Beijing Time, UTC+8) The waterbody morphology, concrete shoreline structure, and vegetation configuration are heavily managed through municipal engineering anthropogenic The surrounding terrestrial vegetation mainly consists of cultivated urban landscaping trees (e.g., *Populus tomentosa*, *Salix babylonica* and *Acer truncatum*) and lawns (*Zoysia tenuifolia*) while native aquatic macrophytes are extremely sparse and fragmented due to periodic waterbody management. Food resources for swans in this site are heavily supplemented by regular artificial feeding (e.g., corn and bread provided by park staff and visitors). Nevertheless, due to thermally stable water circulation and continuous food availability, WPKC has supported a stable overwintering population of approximately 400 whooper swans annually for over a decade, serving as a prominent component of the winter urban ecological landscape in Korla City.

### 2.2. Sample Collection

All samples consisted of fresh feces collected from wild whooper swans. Although other sympatric waterfowl (e.g., *Tadorna ferruginea*) were present in both areas, whooper swan fecal droppings were unambiguously identified based on focal observations of monospecific swan flocks using spotting scopes, combined with their distinctly larger physical size and cylindrical morphology. Field sampling was conducted along the shorelines of core water areas based on spatial distribution and site accessibility. Fecal droppings were gathered immediately after a flock relocated from a specific roosting or foraging microsite. To minimize the possibility of repeated sampling from the same individual, collected droppings were separated by a minimum spatial distance of 5–10 m. Only fresh, moist fecal droppings deposited within 15 min were targeted. To prevent environmental contamination from soil, sediment, or water microbes, only the upper portion of each fecal mass without substrate contact was collected using sterile forceps into sterile cryogenic tubes.

In December 2025 (the peak overwintering period), a total of 34 fresh fecal samples were collected (21 from SBHC and 13 from WPKC). The unequal sample sizes between sites were attributed to field accessibility constraints, ethical guidelines for noninvasive wildlife monitoring, and the localized movement patterns of the flocks during the collection window. All samples were transported to the laboratory in portable ice boxes immediately after collection, and subsequently stored at −80 °C until further analysis.

### 2.3. DNA Extraction and 16S rRNA Gene Sequencing

Following the manufacturer’s protocol, total genomic DNA was isolated from the samples utilizing the E.Z.N.A.^®^ Soil DNA Kit (Omega Bio-tek, Norross, GA, USA). The concentration and purity of the extracted DNA were determined via a NanoDrop 2000 spectrophotometer (Thermo Scientific, Waltham, MA, USA), whereas its quality was verified through 1% agarose gel electrophoresis. Amplification of the bacterial 16S rRNA gene targeting the V3–V4 hypervariable regions was performed employing the specific primers 338F (5′-ACTCCTACGGGAGGCAGCAG-3′) and 806R (5′-GGACTACHVGGGTWTCTAAT-3′). The resulting PCR products were checked on a 2% agarose gel and subsequently isolated with the AxyPrep DNA Gel Extraction Kit (Axygen Biosciences, Union City, CA, USA) prior to elution using Tris-HCl buffer. Quantification of the refined PCR products was conducted with a Qubit 4.0 fluorometer (Thermo Fisher Scientific, Waltham, MA, USA). Subsequently, sequencing libraries were generated in accordance with standard Illumina preparation guidelines, encompassing adapter ligation, magnetic bead purification, PCR enrichment, and the production of single-stranded DNA. Ultimate paired-end sequencing (PE300) was executed utilizing the Illumina MiSeq platform.

### 2.4. Bioinformatic Processing and Statistical Analyses

Raw sequence reads underwent quality filtering and merging before downstream processing. Operational taxonomic units (OTUs) were resolved based on a 97% identity threshold employing the Vsearch program (v2.22.1) to construct the OTU profile. The 97% identity threshold was selected to facilitate direct comparability with the historical gut microbiota literature on migratory waterfowl. For taxonomic assignment, representative OTU sequences were aligned against the Silva 138.1 reference database using the RDP Classifier (v2.13) with a confidence cutoff of 0.7. Given the resolution limitations of partial 16S rRNA gene sequencing, species or strain level designations were avoided, and taxonomic comparisons were focused on the genus level and above. Taxa unclassified at specific levels were retained under their highest assigned rank. To account for variations in sequencing depth, OTU abundances were normalized by subsampling to the minimum sample depth prior to diversity evaluations. Dominant taxa were identified as the top 10 phyla and top 30 genera based on mean relative abundance, while unclassified sequences were retained and categorized as unclassified taxa. Alpha diversity indices of the intestinal microbiota, including Chao1, observed species, Shannon, and Simpson, were evaluated across groups using the non-parametric Wilcoxon rank-sum test. Additionally, the Firmicutes/Bacteroidota (F/B) ratio was calculated for individual samples to assess baseline host energy acquisition and metabolic capacity, with inter-group differences evaluated using the non-parametric Wilcoxon rank-sum test. Beta diversity was evaluated utilizing both weighted and unweighted UniFrac distance metrics, followed by visualization via principal coordinate analysis (PCoA), and statistically tested for significance using Permutational Multivariate Analysis of Variance (PERMANOVA via adonis2 with 999 permutations). Differences in microbial taxonomic abundance between groups were statistically evaluated using the Kruskal–Wallis rank-sum test with a subsequent Dunn’s post hoc test. To identify specific microbial biomarkers distinct to each habitat, linear discriminant analysis effect size (LEfSe) was performed, where taxa displaying *p* < 0.05 and a logarithmic LDA score > 3.0 were deemed significant. Furthermore, functional profiles of the gut microbial communities were forecasted using PICRUSt2 (v2.5.2) with Nearest Sequenced Taxon Index (NSTI) evaluation, and the predicted metabolic pathways were characterized based on the Kyoto Encyclopedia of Genes and Genomes (KEGG) resource. Inter-group variations in functional pathways were verified through Welch’s *t*-test with Benjamini–Hochberg correction (q < 0.05) and Kruskal–Wallis tests combined with LEfSe analysis (*p* < 0.05). All computational analyses and visualizations were conducted in R (v4.5.1). Community management, beta-diversity matrices, and PCoA plots were generated with phyloseq (v1.44.0), while alpha-diversity statistics and PERMANOVA tests (adonis2, 999 permutations) were executed using vegan (v2.6-4). Group-wise statistical comparisons relied on Kruskal–Wallis tests via coin (v1.4-3) followed by Dunn’s post hoc pairwise comparisons, with heatmaps rendered using pheatmap (v1.0.12). Differentially abundant taxa were identified through LEfSe analysis based on a logarithmic LDA threshold > 3.0 (*p* < 0.05). For PICRUSt2 functional predictions, differences in KEGG pathway abundances between groups were evaluated by Welch’s *t*-test, adjusting for multiple testing with the Benjamini–Hochberg FDR method (*q* < 0.05).

## 3. Results

### 3.1. Sequencing Data and Operational Taxonomic Unit (OTU) Characteristics

High-throughput 16S rRNA gene sequencing generated a total of 3,222,466 raw read pairs across the 34 fecal samples (ranging from 53,445 × 2 to 130,165 × 2 read pairs per sample). After quality filtering and chimera removal, a total of 3,042,471 high-quality retained reads were obtained, with 51,084 to 122,888 reads per sample (mean ± SD: 89,484 ± 22,251 reads per sample; average retention rate: 94.40%). The average Q30 quality score reached 95.12%, demonstrating robust sequencing depth and high data quality across all samples (Supplementary Table S1).

**Figure 3:**
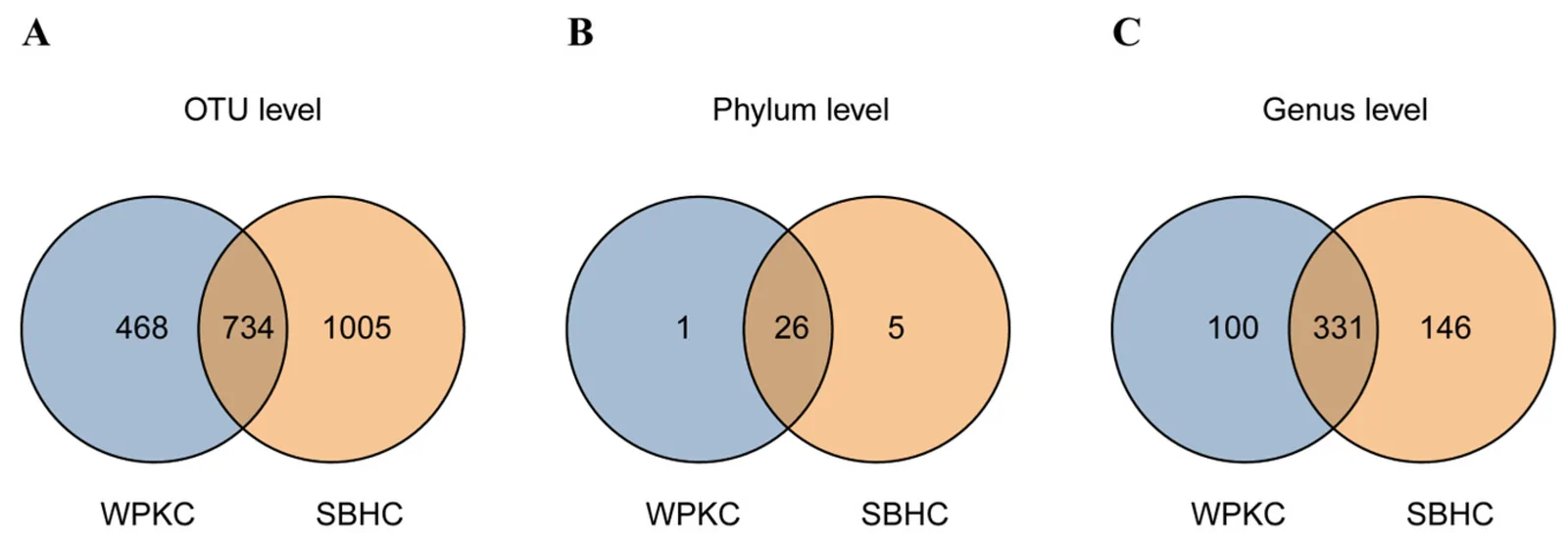
Venn diagram. The Venn diagrams show the numbers of OTUs (97% sequence identity) (**A**), phyla (**B**) and genera (**C**) that were shared or not shared by Swan Bay in Hejing County (SBHC) and Workers’ Park in Korla City (WPKC) individuals depending on overlaps.

Based on 16S rRNA high-throughput sequencing, a total of 2207 operational taxonomic units (OTUs) were identified at the 97% sequence similarity threshold, with an average of 229 ± 153 OTUs per sample. These OTUs were taxonomically assigned to 32 phyla, 84 classes, 181 orders, 304 families, 577 genera, and 362 species. Rarefaction curve analysis demonstrated that all curves gradually approached saturation with increasing sequencing depth (Supplementary Figure S1), indicating that the sequencing effort was sufficient to capture the majority of bacterial diversity within the samples and that additional sequencing would contribute minimally to further diversity recovery. Gut microbiota of swans from the two overwintering habitats shared 734 OTUs at the OTU level (Figure 3A), suggesting the existence of a conserved core microbiota among individuals inhabiting different overwintering environments. At higher taxonomic levels, the two groups shared 26 bacterial phyla (Figure 3B) and 331 genera (Figure 3C), further indicating that the gut microbial communities of Cygnus cygnus maintained a relatively stable core community structure despite habitat differences.

### 3.2. Alpha Diversity Analysis

**Figure 4:**
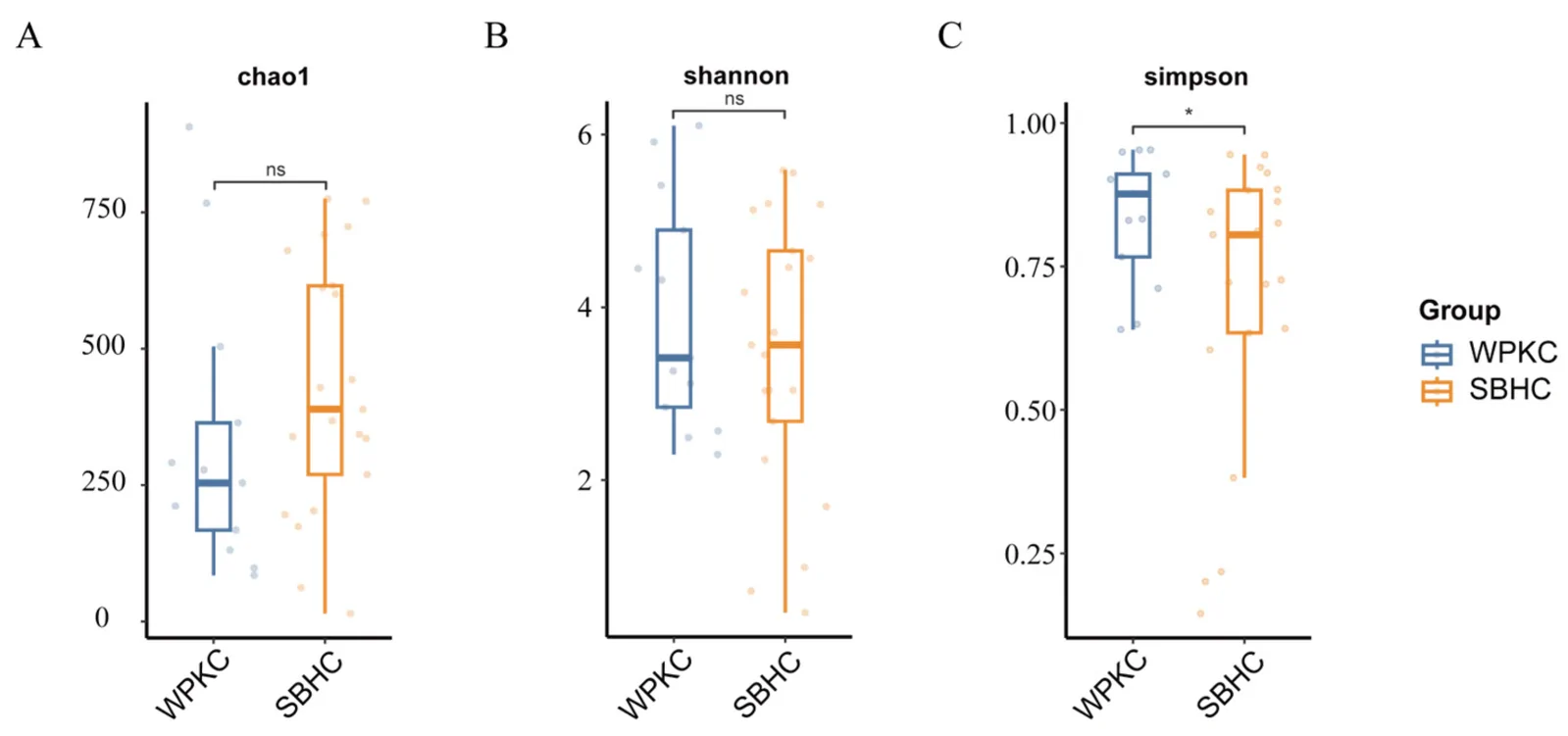
Alpha diversity between WPKC and SBHC: (**A**) Chao1 Index; (**B**) Shannon Index; (**C**) Simpson Index. Note: Asterisks indicate statistically significant differences between groups (* *p* < 0.05); ns, not significant (*p* > 0.05).

Alpha diversity analysis revealed differences in microbial richness and evenness between the two overwintering groups (Figure 4). Specifically, the Simpson index was significantly higher in the WPKC group than in the SBHC group (*p* < 0.05), suggesting that swans wintering in the urban wetland habitat harbored a more even and diverse gut microbial community. In contrast, no significant differences were detected between the two groups in the Chao1 or Shannon indices.

### 3.3. Beta Diversity Analysis

**Figure 5:**
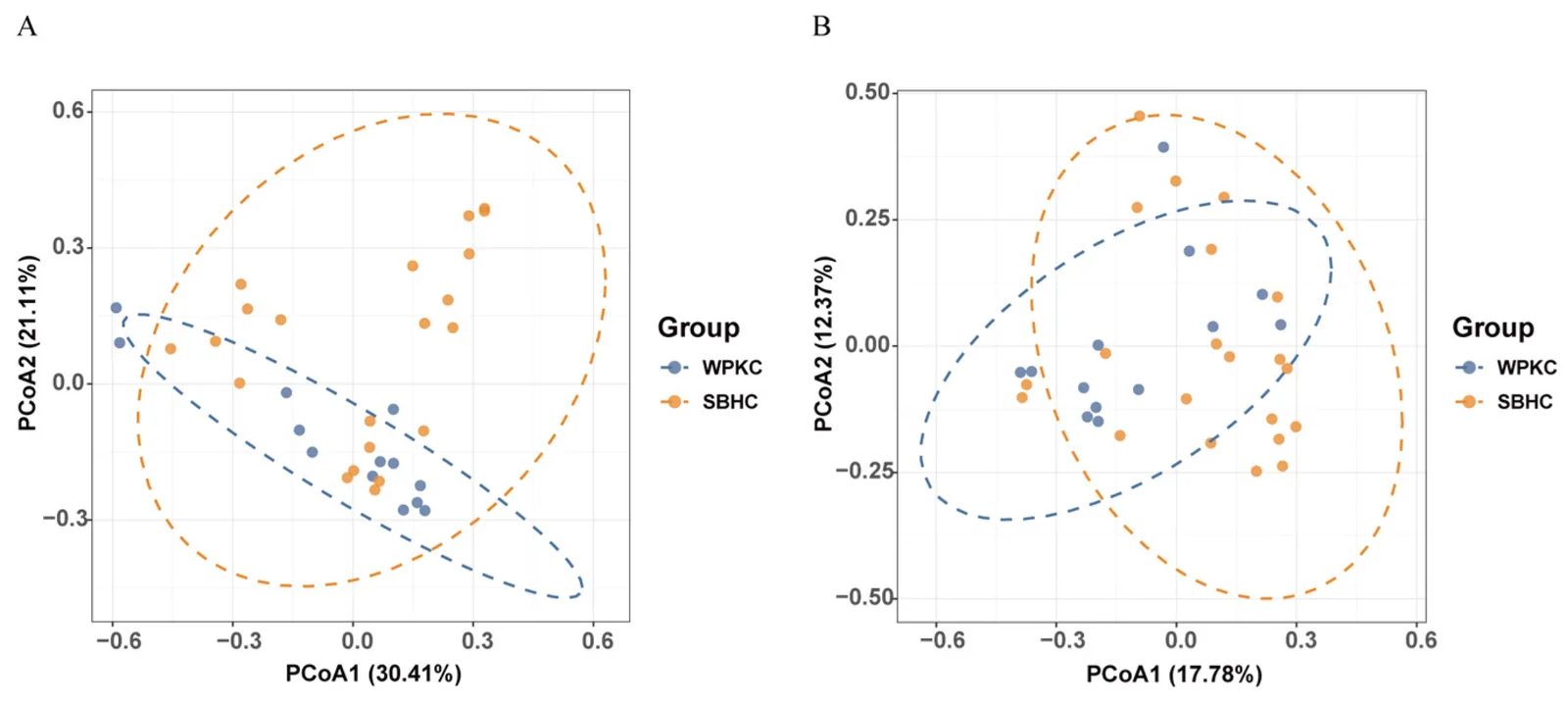
β diversity: (**A**) Weighted UniFrac. (**B**) Unweighted UniFrac.

Principal coordinate analysis (PCoA) based on weighted and unweighted UniFrac distance matrices revealed partial overlap between samples from the two groups, although a tendency toward clustering by habitat type was observed (Figure 5). Permutational multivariate analysis of variance (PERMANOVA, 999 permutations) based on weighted UniFrac (R^2^ = 0.0944, *p* = 0.003) and unweighted UniFrac (R^2^ = 0.0606, *p* = 0.006) distance matrices further confirmed that gut microbial community structures differed significantly between the two overwintering habitats. These results suggest that habitat environment is a key factor shaping the gut microbiota composition of swans.

### 3.4. Composition of the Gut Microbial Community

**Figure 6:**
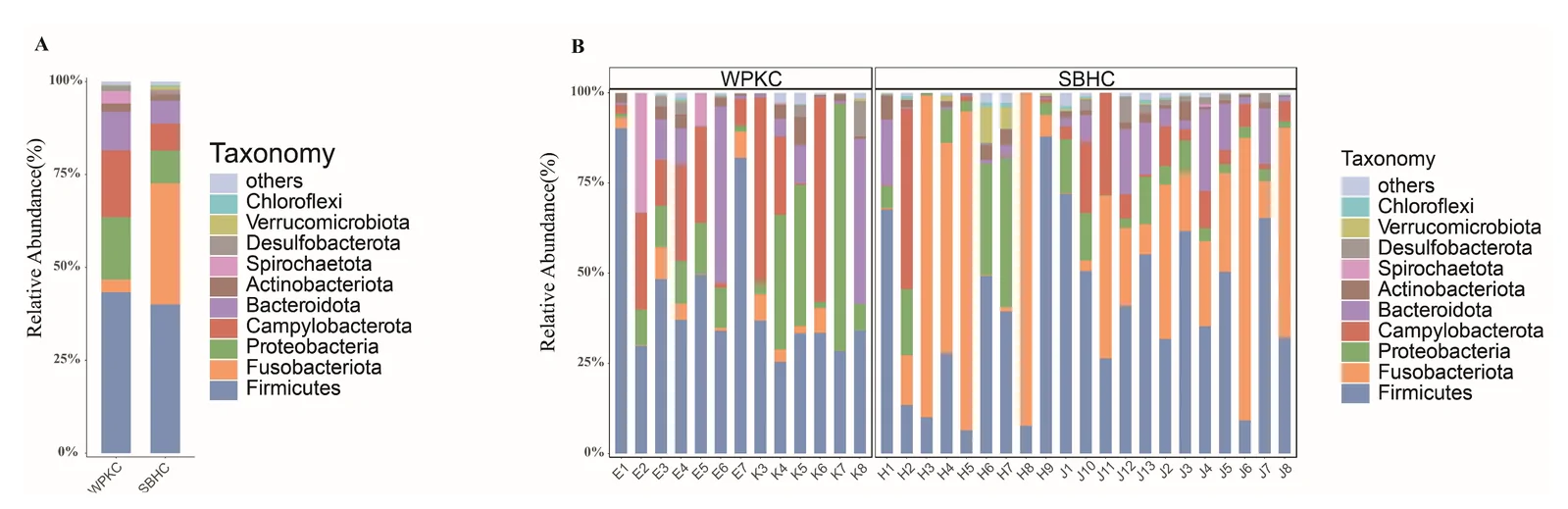
Relative abundance of the top 10 bacterial phyla in the gut microbiota of Whooper Swans (*Cygnus cygnus*). (**A**) Accumulated relative abundance grouped by overwintering habitats (WPKC: urban artificial wetland; SBHC: natural riverine wetland). (**B**) Relative abundance profiles across individual samples within each habitat.

At the phylum level, the gut microbial communities of both groups were dominated by Firmicutes, *Fusobacteriota*, Proteobacteria, Campylobacterota, and Bacteroidota, although their relative abundances differed markedly between habitats (Figure 6). In the WPKC group, Firmicutes was the dominant phylum, accounting for 43.30% of the total relative abundance, followed by Campylobacterota (17.99%), Proteobacteria (16.78%), and Bacteroidota (10.39%). *Fusobacteriota* and Spirochaetota accounted for 3.44% and 3.30%, respectively, whereas the remaining phyla were present at relatively low abundances. Similarly, Firmicutes was also the dominant phylum in the SBHC group, with an average relative abundance of 40.05%, followed by *Fusobacteriota* (32.57%). Together, these two phyla constituted the major components of the gut microbiota in this group. Proteobacteria (8.82%), Campylobacterota (7.29%), and Bacteroidota (6.12%) were also abundant, while the remaining phyla each accounted for less than 2.00%.

Overall, although the two groups exhibited similar core microbial compositions at higher taxonomic levels, substantial shifts in the relative abundances of dominant phyla were observed, reflecting habitat-specific structural variation in the gut microbiota.

### 3.5. Community Composition at the Genus Level

**Figure 7:**
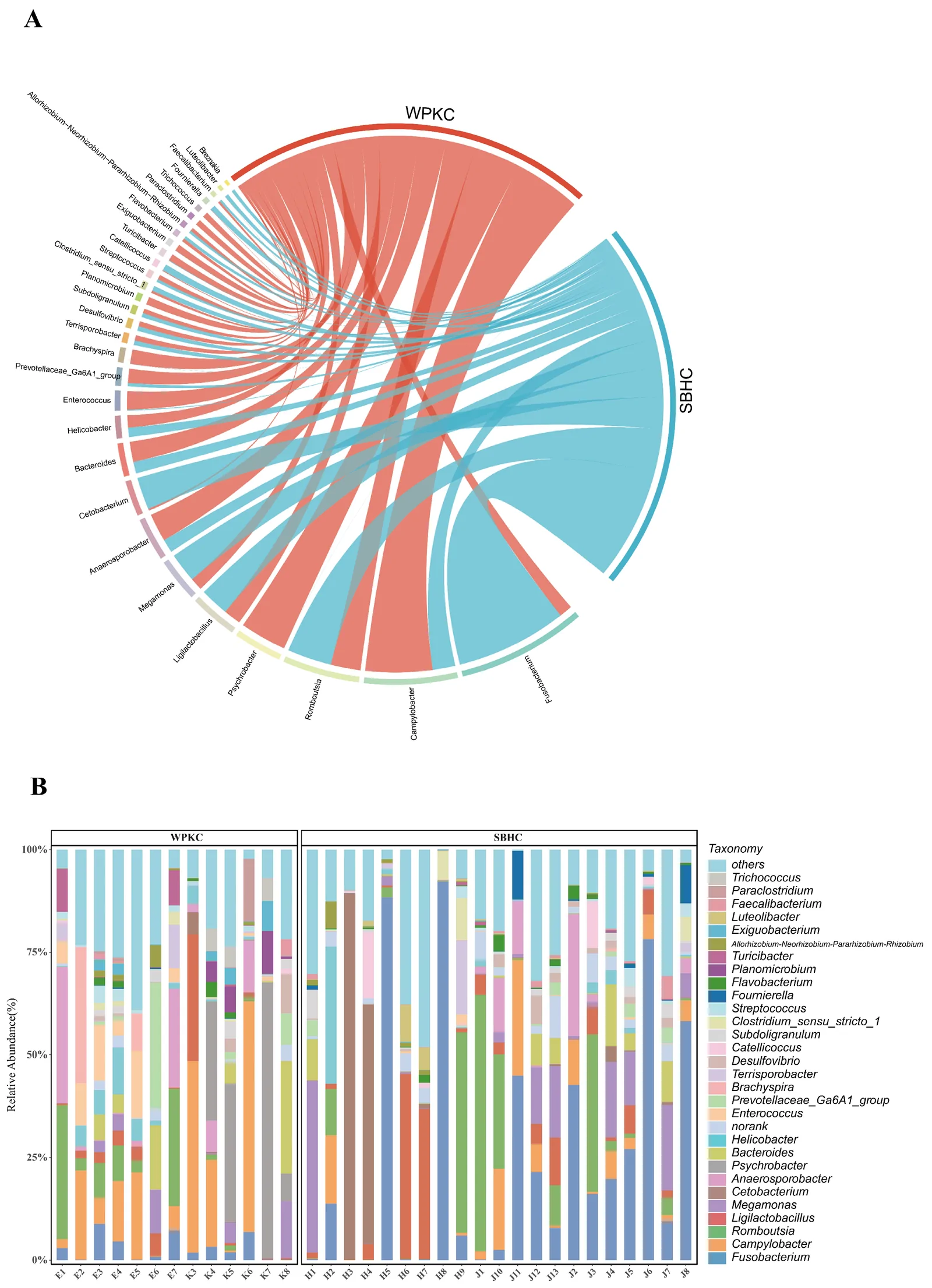
Top 30 genus-level relative abundance. (**A**) Groups. (**B**) Samples.

At the genus level, more pronounced differences in gut microbial composition were detected between the two groups (Figure 7). In the WPKC group, *Campylobacter* was the dominant genus, with an average relative abundance of 15.15%, followed by *Psychrobacter* (10.53%), *Romboutsia* (6.76%), *Anaerosporobacter* (6.37%), *Bacteroides* (4.59%), *Enterococcus* (4.17%), and Ligilactobacillus (3.92%). In addition, genera including *Brachyspira*, *Prevotellaceae* Ga6A1 group, *Fusobacterium*, *Megamonas*, and *Helicobacter* were detected at moderate abundances. In contrast, the SBHC group was characterized by a predominance of *Fusobacterium*, with a relative abundance of 25.23%, followed by *Romboutsia* (10.02%), *Cetobacterium* (7.32%), *Megamonas* (6.68%), *Ligilactobacillus* (6.51%), and *Campylobacter* (5.14%). Genera such as *Anaerosporobacter*, *Bacteroides*, and *Helicobacter* also contributed substantially to the microbial community. Collectively, the WPKC group was characterized by relatively higher abundances of *Campylobacter* and *Psychrobacter*, whereas the SBHC group exhibited enrichment of *Fusobacterium*, *Romboutsia*, and *Cetobacterium*, suggesting substantial habitat-associated differentiation in gut microbial composition at the genus level.

### 3.6. LEfSe Analysis of Differentially Abundant Taxa

**Figure 8:**
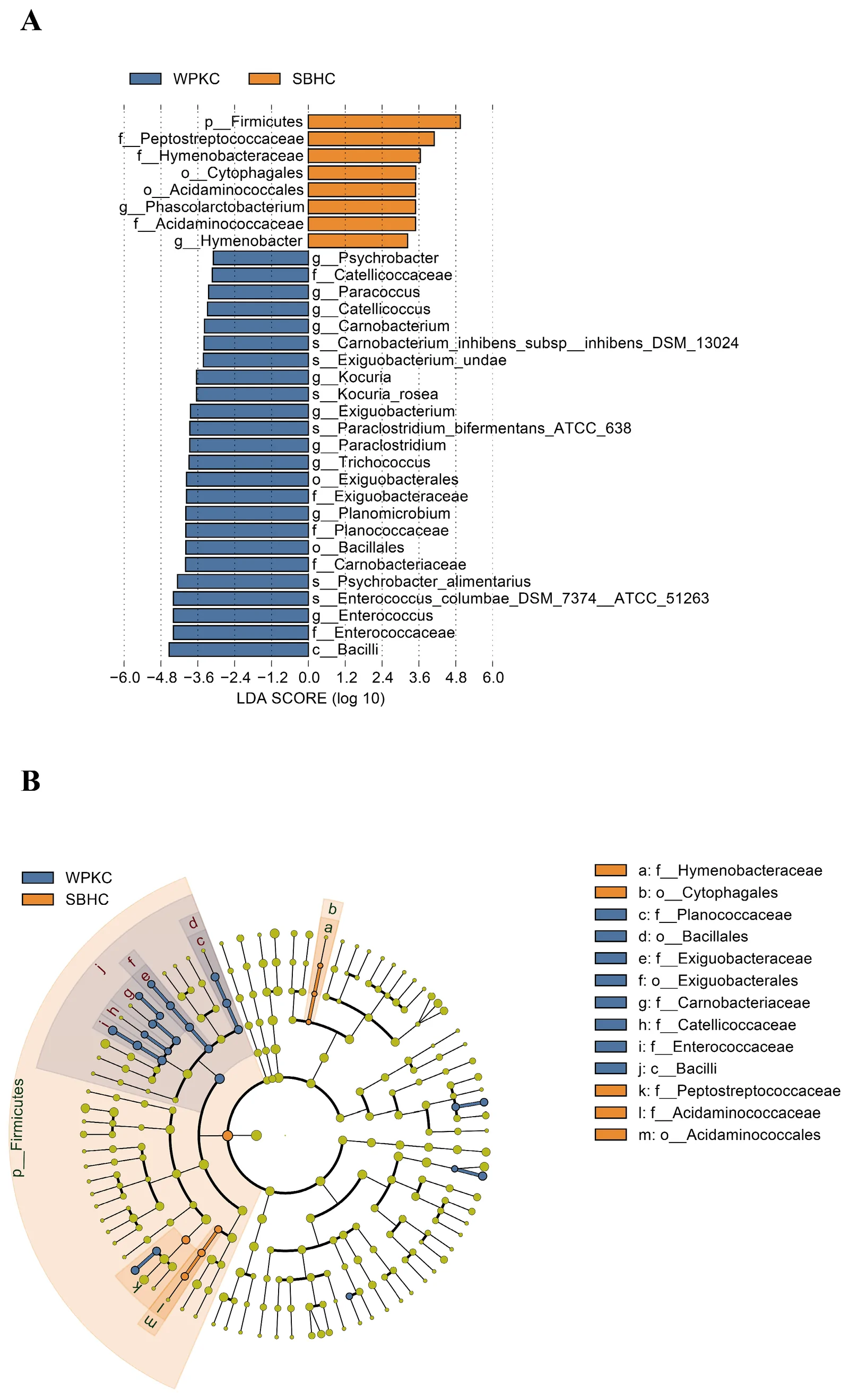
LEfSe analysis of the gut microbiota in whooper swans. (**A**) LDA score results; (**B**) LEfSe cladogram.

LEfSe analysis identified multiple significantly enriched bacterial taxa between the two groups (logarithmic LDA score > 3.0, *p* < 0.05) (Figure 8). Overall, the WPKC group contained a greater number of differentially enriched biomarker taxa than the SBHC group (Figure 8A). In the WPKC group, key biomarkers were predominantly enriched within the class Bacilli, including *Enterococcaceae* (e.g., *Enterococcus* and *Enterococcus columbae* DSM 7374 ATCC 51263), *Carnobacteriaceae* (e.g., *Carnobacterium* and *Carnobacterium inhibens*), *Planococcaceae* (e.g., *Planomicrobium*), *Exiguobacteraceae* (e.g., *Exiguobacterium* and *Exiguobacterium undae*), and *Catellicoccaceae* (e.g., *Catellicoccus*). Additional significantly enriched genera in WPKC included *Psychrobacter* (along with *Psychrobacter alimentarius*), *Paraclostridium* (along with *Paraclostridium bifermentans* ATCC 638), *Paracoccus*, *Kocuria* (along with Kocuria rosea), and *Trichococcus*. By comparison, fewer biomarker taxa were enriched in the SBHC group, which were mainly characterized by the phylum Firmicutes (LDA > 4.8), *Peptostreptococcaceae*, *Acidaminococcaceae* (and the order Acidaminococcales), *Phascolarctobacterium*, as well as *Hymenobacteraceae* (and *Hymenobacter*) affiliated with the order Cytophagales.

The LEfSe cladogram further illustrates the taxonomic hierarchical distribution of these differential biomarkers from phylum to species level between the two habitats (Figure 8B). Together, these results highlight clear habitat-specific microbial enrichment profiles and differential biomarker taxa between the two overwintering swan populations.

### 3.7. PICRUSt2 Functional Prediction Analysis

**Figure 9:**
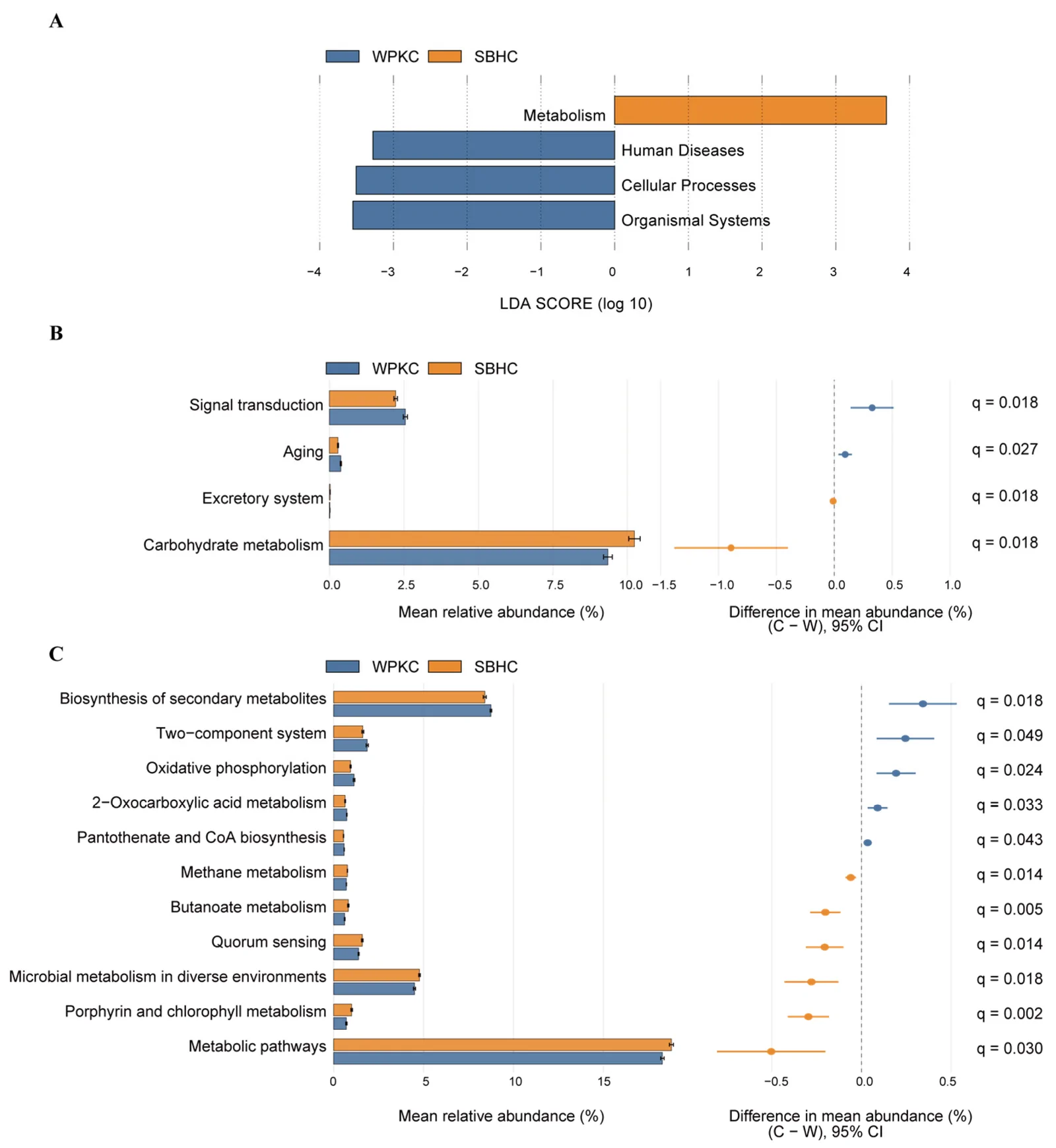
PICRUSt-predicted differences in KEGG functional pathways in the gut microbiota of whooper swans. (**A**) KEGG level 1, (**B**) KEGG level 2, and (**C**) KEGG level 3.

PICRUSt2-based functional prediction demonstrated significant differences in multiple KEGG functional categories between the two groups (Figure 9). At KEGG level 1, the SBHC group showed significant enrichment in the category “Metabolism,” whereas the WPKC group exhibited relatively higher enrichment in “Human Diseases,” “Cellular Processes,” and “Organismal Systems” (Figure 9A). At KEGG level 2, four significantly different functional pathways were identified (Welch’s *t*-test with BH correction, q < 0.05). Specifically, the SBHC group was significantly enriched in “Excretory system” and “Carbohydrate metabolism,” while the WPKC group showed enrichment in “Signal transduction” and “Aging”-related pathways (Figure 9B). Further analysis at KEGG level 3 identified 11 significantly differentially abundant pathways with relative abundances exceeding 0.5% (q < 0.05) (Figure 9C). The SBHC group was significantly enriched in pathways associated with “Porphyrin and chlorophyll metabolism,” “Butanoate metabolism,” “Methane metabolism,” “Quorum sensing,” “Microbial metabolism in diverse environments,” and “Metabolic pathways.” In contrast, the WPKC group exhibited enrichment in pathways related to “Biosynthesis of secondary metabolites,” “Oxidative phosphorylation,” “2-Oxocarboxylic acid metabolism,” “Pantothenate and CoA biosynthesis,” and “Two-component system.” Overall, the functional pathways enriched in the SBHC group were primarily associated with carbon metabolism, short-chain fatty acid metabolism, and microbial metabolic processes in complex environments, whereas the WPKC group displayed functional characteristics more closely related to energy metabolism regulation, signal transduction, and secondary metabolite biosynthesis.

## 4. Discussion

This study comparatively investigated the gut microbial composition and predicted functional characteristics of whooper swans (*Cygnus cygnus*) overwintering in two contrasting habitats in Xinjiang, China, namely a natural riverine wetland and an urban artificial wetland. Overall, although the two groups exhibited similar core microbial compositions at higher taxonomic levels, substantial shifts in the relative abundances of dominant phyla were observed, reflecting habitat-specific structural variation in the gut microbiota. Concurrently, these findings demonstrate distinct microbial enrichment profiles associated with different overwintering habitats, suggesting that environmental exposure and site-specific diets strongly shape the differential representation of key biomarker taxa in the swan gut microbiota. Consistent with our initial hypothesis, the results suggested that overwintering habitat type was potentially associated with gut microbial diversity, community structure, and functional profiles in migratory swans. Specifically, we hypothesized that (1) urban wetland environments would correlate with increased microbial diversity and environmental microbial exposure due to intensified anthropogenic disturbance and heterogeneous food resources, and (2) natural riverine habitats would retain microbial assemblages more closely associated with natural nutrient metabolism and aquatic ecological adaptation. The observed patterns largely supported these hypotheses.

Alpha diversity analysis revealed that only the Simpson index was significantly higher in the urban wetland group than in the natural riverine group, suggesting that urbanization primarily influenced microbial evenness and dominance structure rather than overall taxonomic richness in swans inhabiting urban environments. Gut microbial alpha diversity is generally considered an important indicator of microbial ecosystem stability and host adaptive potential [20]. Previous studies have frequently reported higher gut microbial diversity in wild animals than in captive individuals [21,22]. However, increasing evidence suggests that animals exposed to highly heterogeneous anthropogenic environments may also exhibit elevated microbial diversity due to increased environmental microbial inputs and diversified dietary resources [23,24,25]. Similar patterns have been observed in urban populations of white-crowned sparrows (*Zonotrichia leucophrys*), where urban-associated habitats promoted higher gut microbial diversity [26]. Therefore, the elevated diversity observed in the urban wetland group may reflect the combined effects of anthropogenic food supplementation, increased habitat heterogeneity, and greater exposure to environmental microorganisms associated with urban ecosystems [27]. These findings support our first hypothesis that urbanized overwintering habitats reshape the gut microbial diversity of migratory waterbirds.

Beta diversity analysis revealed significant differences in microbial community structure between the two overwintering groups, although partial overlap remained in ordination space. Importantly, given the observational nature of this field study, these beta-diversity patterns reflect an association between habitat types and microbiota structure rather than a definitive causal relationship. This pattern suggests that habitat-related environmental variation is associated with shifted relative abundance structure of microbial communities rather than completely replacing the host-associated microbiota. Similar observations have been reported in other migratory Anatidae species, including swan geese (*Anser cygnoides*) [9]. The persistence of overlapping microbial communities further may be consistent with the hypothesis that host phylogeny and shared physiological characteristics continue to maintain a conserved core microbiota despite environmental differences. Such findings support the hypothesis that avian gut microbiota may be jointly influenced by host physiological baseline and site-specific environmental exposure.

At the phylum level, both groups were dominated by Firmicutes, *Fusobacteriota*, Proteobacteria, Campylobacterota, and Bacteroidota, and a high proportion of shared OTUs and bacterial phyla was observed between habitats. These results further support the existence of a relatively conserved core microbiota within the same host species. Similar “core microbiota plus environmentally responsive taxa” patterns have been reported in multiple bird species [28,29,30]. In line with previous studies [1,2], among these dominant phyla, Firmicutes, Proteobacteria, and Bacteroidota served as the core bacterial groups in our subjects and are primarily involved in carbohydrate degradation, nutrient metabolism, and host energy acquisition. Their predominance is consistent with the PICRUSt2 functional predictions showing a high proportion of metabolism-related pathways in both groups.

Despite this conserved microbial framework, notable differences in relative abundance patterns were observed between habitats. The SBHC group exhibited a markedly higher abundance of *Fusobacteriota*, particularly *Cetobacterium*-related taxa. Previous studies have shown that *Fusobacteriota* commonly dominate the intestinal microbiota of aquatic organisms [31,32]. This enrichment may potentially reflect an indirect association with aquatic environments and natural food resources in the riverine wetland. Previous studies have also suggested that swans may shift their diet composition during winter when plant resources become limited [33]. Therefore, we hypothesize a potential link between *Fusobacteriota*/*Cetobacterium* enrichment and dietary variation and speculate that swans in natural overwintering habitats may broaden their dietary niches or exploit alternative aquatic-associated food resources to compensate for winter energy constraints. However, we emphasize that these dietary links remain purely hypothetical. Definitive validation will require future empirical investigations using stable isotope analysis, fecal dietary metabarcoding, and metagenomic approaches.

In contrast, the urban wetland group exhibited significant enrichment of opportunistically pathogenic and environmental biomarker taxa, particularly *Enterococcus* (including *Enterococcus columbae*) and *Paraclostridium* (Figure 7). *Enterococcus* is widely recognized as a ubiquitous gut commensal with strong opportunistic pathogenic potential in wild birds, often associated with degraded water quality and anthropogenic pollution [34,35,36,37]. Its marked enrichment in urban wetland swans suggests that urban environments may elevate exposure to environmental pathogens and anthropogenic microbial sources. Its enrichment in urban wetland swans suggests that urban environments may increase exposure to environmental pathogens and anthropogenic microbial sources. In urban wetlands, intensive overlap between human recreational activities and bird habitats likely increases opportunities for cross-species microbial transmission [38,39,40]. This finding further supports our second hypothesis that urban habitats may elevate potential pathogen exposure and alter microbial ecological niches in migratory birds. These observations highlight the need for future longitudinal studies to evaluate whether pathogen exposure in urban habitats translates into elevated health risks for migratory waterbirds and potential zoonotic implications.

The Firmicutes/Bacteroidota (F/B) ratio is commonly regarded as an indicator of host metabolic status and energy acquisition efficiency [41]. Previous studies have shown that captivity often substantially alters the F/B ratio in wildlife gut microbiota [24,25]. However, no significant difference in the F/B ratio was detected between the urban wetland and natural riverine groups in the present study. This finding suggests that the effects of urbanization on the gut microbiota of whooper swans cannot simply be equated with typical captivity-associated microbial restructuring. Although urban wetlands modify food availability and environmental exposure patterns, swans in urban habitats still retain substantial natural foraging behavior and mobility. Therefore, microbial changes in urban swans likely represent an ecological adjustment rather than a complete transition toward a captivity-associated microbiota profile. Our findings partially align with previous observations by Wang et al. [8], who demonstrated that local environmental pressures exert a strong driving force on waterbird gut microbiota structure. Consistent with their findings, beta-diversity separation and the differential enrichment of environmental biomarkers (such as Psychrobacter) highlight the high plasticity of whooper swan gut microbiota in response to site-specific ecological niches. However, notable discrepancies also emerge regarding metabolic indicators. While Wang et al. reported dramatic shifts in the Firmicutes/Bacteroidota (F/B) ratio across geographically distant wintering grounds (e.g., inland vs. coastal wetlands), no significant difference in the F/B ratio was observed between our urban and natural wetland groups. This divergence suggests that localized microhabitat alterations within the same overwintering region primarily induce functional microbial fine-tuning rather than the profound systemic restructuring associated with broad geographical gradients.

At the genus level, the SBHC group exhibited relatively higher abundances of Romboutsia, Megamonas, and *Ligilactobacillus*. Previous studies have suggested that Romboutsia possesses strong carbohydrate metabolic potential [42], Megamonas is associated with carbohydrate fermentation and short-chain fatty acid production [43], and *Ligilactobacillus* contributes to lactate production and intestinal homeostasis maintenance [44,45]. Additionally, *Phascolarctobacterium*, enriched in the SBHC group, is closely associated with propionate metabolism and short-chain fatty acid production [46,47]. Combined with the enrichment of metabolism-related pathways identified through PICRUSt2 analysis, these findings suggest that gut microbiota in natural overwintering habitats may be more strongly associated with nutrient metabolism and host energy acquisition, thereby supporting adaptation to energetically demanding winter conditions.

Conversely, Psychrobacter and Enterococcus were significantly enriched in the urban wetland group. Psychrobacter is commonly associated with low-temperature environments [48] and has frequently been reported in overwintering waterbirds [49]. Its enrichment in the urban group may reflect increased exposure to frozen surfaces, artificial water systems, and cold urban microhabitats. Meanwhile, although Enterococcus is a common intestinal commensal bacterium, certain strains possess opportunistic pathogenic potential [50,51]. The enrichment of these taxa suggests that urban wetland environments may reshape microbial ecological niches beyond baseline metabolic functions and potentially increase health-related microbial risks in migratory swans. Importantly, the present study relied on 16S rRNA gene sequencing and PICRUSt2-based functional prediction, which estimate the potential functional capacity of microbial communities rather than directly measuring actual metabolic activity. Therefore, future studies integrating metagenomics, metatranscriptomics, metabolomics, and stable isotope analyses will be necessary to clarify the mechanistic links between overwintering habitat conditions, microbial functional activity, and host physiological adaptation.

Admittedly, this study has limitations associated with non-invasive sampling of free-ranging populations. The inability to assign collected fecal specimens to specific individuals precluded us from accounting for host-centric variables, including sex, age class, and body condition. Given that host physiology can substantially modulate gut microbiota composition and metabolic output, these unmeasured host traits represent potential sources of inter-individual variation in our dataset. Future empirical studies combining GPS-telemetry tracking, host molecular sexing, and long-term behavioral observations will provide higher resolution into how individual host states interact with anthropogenic disturbance to shape gut microbial dynamics.

## 5. Conclusions

Using 16S rRNA gene high-throughput sequencing, this study evaluated the gut microbial profiles of whooper swans (*Cygnus cygnus*) overwintering in natural riverine wetlands and urban artificial wetlands in Xinjiang, China. Our findings suggest that while whooper swans across both habitats share broad taxonomic features, distinct habitat-associated microbial variation exists. Swans inhabiting natural riverine habitats displayed microbial profiles enriched in taxa typically associated with natural nutrient fermentation, whereas those in urban artificial wetlands exhibited higher community evenness and an increased representation of environmentally associated taxa and potential opportunistic bacterial lineages. These differences suggest potential associations between overwintering habitat environments and the gut microbial structure of migratory waterbirds. Importantly, future studies incorporating multi-omics approaches (e.g., metagenomics and dietary metabarcoding) and direct pathogen isolation are needed to verify whether these microbial shifts affect host physiological health or present zoonotic transmission risks. Overall, this work provides preliminary baseline data for understanding the microbial ecology of migratory waterbirds in contrasting overwintering landscapes.

## Data Availability

The data presented in this study are available on request from the corresponding author. The raw data (including original images and underlying numerical values for all figures) are stored by the authors.

## Supplementary Materials

The following supporting information can be downloaded at: https://www.mdpi.com/article/10.3390/ani16172638/s1.

## Abbreviations

The following abbreviations are used in this manuscript:

WPKC	Workers’ Park in Korla City
SBHC	Swan Bay in Hejing County
OTUs	Operational Taxonomic Units
PCoA	Principal Coordinate Analysis
LEfSe	Linear Discriminant Analysis Effect Size
KEGG	Kyoto Encyclopedia of Genes and Genomes

## References

[1] Bodawatta K.H., Hird S.M., Grond K., Poulsen M., Jønsson K.A. (2022). Avian gut microbiomes taking flight. Trends Microbiol..

[2] Grond K., Sandercock B.K., Jumpponen A., Zeglin L.H. (2018). The avian gut microbiota: Community, physiology and function in wild birds. J. Avian Biol..

[3] Sun F., Chen J., Liu K., Tang M., Yang Y. (2022). The avian gut microbiota: Diversity, influencing factors, and future directions. Front. Microbiol..

[4] Gao L., Liu L., Du C., Hou Q. (2021). Comparative analysis of fecal bacterial microbiota of six bird species. Front. Vet. Sci..

[5] Somers S.E., Davidson G.L., Johnson C.N., Reichert M.S., Crane J.M.S., Ross R.P., Stanton C., Quinn J.L. (2023). Individual variation in the avian gut microbiota: The influence of host state and environmental heterogeneity. Mol. Ecol..

[6] Sun M., Halimubieke N., Fang B., Valdebenito J.O., Xu X., Sheppard S.K., Székely T., Zhang T., He S., Lu R. (2024). Gut microbiome in two high-altitude bird populations showed heterogeneity in sex and life stage. FEMS Microbes.

[7] Murray M.H., Lankau E.W., Kidd A.D., Welch C.N., Ellison T., Adams H.C., Lipp E.K., Hernandez S.M. (2020). Gut microbiome shifts with urbanization and potentially facilitates a zoonotic pathogen in a wading bird. PLoS ONE.

[8] Wang W., Huang S., Yang L., Zhang G. (2021). Comparative analysis of the fecal bacterial microbiota of wintering whooper swans (*Cygnus cygnus*). Front. Vet. Sci..

[9] Wu Y., Yang Y., Cao L., Yin H., Xu M., Wang Z., Liu Y., Wang X., Deng Y. (2018). Habitat environments impacted the gut microbiome of long-distance migratory swan geese but central species conserved. Sci. Rep..

[10] Liu G., Xu N., Yu C. (2024). Comparative analysis of the microbiome of sympatric wintering Bean Geese, Domestic Ducks, humans, and soil at Shengjin Lake of China reveals potential public risk to human health. Avian Res..

[11] Ao P., Wang X., Meng F., Batbayar N., Moriguchi S., Shimada T., Koyama K., Park J., Kim H., Ma M. (2020). Migration routes and conservation status of the Whooper Swan Cygnus cygnus in East Asia. Wildfowl.

[12] Lee J.-Y., Nam H.-K., Park J.-Y., Kang S.-G., Batbayar N., Kim D.-W., Hwang J.-W., Tsend O., Natsagdorj T., Nergui J. (2023). Migration routes and differences in migration strategies of Whooper Swans between spring and autumn. Avian Res..

[13] Shimada T., Yamaguchi N.M., Hijikata N., Hiraoka E., Hupp J.W., Flint P.L., Tokita K.-I., Fujita G., Uchida K., Sato F. (2014). Satellite tracking of migrating Whooper Swans Cygnus cygnus wintering in Japan. Ornithol. Sci..

[14] Li S., Meng W., Liu D., Yang Q., Chen L., Dai Q., Ma T., Gao R., Ru W., Li Y. (2018). Migratory Whooper Swans Cygnus cygnus Transmit H5N1 Virus between China and Mongolia: Combination Evidence from Satellite Tracking and Phylogenetics Analysis. Sci. Rep..

[15] Yang L., Wang W., Sun P., Huang S., Gao R., Kong D., Ru W., Wronski T., Zhang G. (2021). Extrinsic factors, endocrine mechanisms, and behavioral indicators of migratory restlessness in wintering whooper swans (*Cygnus cygnus*). Sci. Rep..

[16] Marra P.P., Studds C.E., Wilson S., Sillett T.S., Sherry T.W., Holmes R.T. (2015). Non-breeding season habitat quality mediates the strength of density-dependence for a migratory bird. Proc. R. Soc. B.

[17] Cooper N.W., Yanco S.W., Rushing C.S., Sillett T.S., Marra P.P. (2024). Non-breeding conditions induce carry-over effects on survival of migratory birds. Curr. Biol..

[18] Kouzov S.A., Kravchuk A.V., Koptseva E.M., Gubelit Y.I., Zaynagutdinova E.M., Abakumov E.V. (2024). Ecological and phylogenetic aspects of the spring diet of three palaearctic species of swans. BMC Ecol. Evol..

[19] Wood K.A., Newth J.L., Hilton G.M., Rees E.C. (2021). Behavioural and energetic consequences of competition among three overwintering swan (*Cygnus* spp.) species. Avian Res..

[20] Éliás A.J., Földvári-Nagy K.C., Al-Gharati Y.Z., Veres D.S., Schnabel T., Teutsch B., Erőss B., Hegyi P., Lenti K., Földvári-Nagy L. (2026). Effect of probiotic supplementation on the gut microbiota diversity in healthy populations: A systematic review and meta-analysis of randomised controlled trials. BMC Med..

[21] Schmidt E., Mykytczuk N., Schulte-Hostedde A.I. (2019). Effects of the captive and wild environment on diversity of the gut microbiome of deer mice (*Peromyscus maniculatus*). ISME J..

[22] San Juan P.A., Castro I., Dhami M.K. (2021). Captivity reduces diversity and shifts composition of the Brown Kiwi microbiome. Anim. Microbiome.

[23] Diaz J., Reese A.T. (2021). Possibilities and limits for using the gut microbiome to improve captive animal health. Anim. Microbiome.

[24] Bensch H.M., Tolf C., Waldenström J., Lundin D., Zöttl M. (2023). Bacteroidetes to Firmicutes: Captivity changes the gut microbiota composition and diversity in a social subterranean rodent. Anim. Microbiome.

[25] Wang Y., Yang X., Zhang M., Pan H. (2023). Comparative Analysis of Gut Microbiota between Wild and Captive Golden Snub-Nosed Monkeys. Animals.

[26] Phillips J.N., Berlow M., Derryberry E.P. (2018). The Effects of Landscape Urbanization on the Gut Microbiome: An Exploration Into the Gut of Urban and Rural White-Crowned Sparrows. Front. Ecol. Evol..

[27] Yan S., Zhang Y., Huang J., Liu Y., Li S. (2024). Comparative Study of Gut Microbiome in Urban and Rural Eurasian Tree Sparrows. Animals.

[28] Cockerham S., Lee B., Orben R.A., Suryan R.M., Torres L.G., Warzybok P., Bradley R., Jahncke J., Young H.S., Ouverney C. (2019). Microbial Ecology of the Western Gull (*Larus occidentalis*). Microb. Ecol..

[29] Berlow M., Phillips J.N., Derryberry E.P. (2021). Effects of Urbanization and Landscape on Gut Microbiomes in White-Crowned Sparrows. Microb. Ecol..

[30] He Y., Zhang M., Dai C., Yu L. (2023). Comparison of the Gut Microbial Communities of Domestic and Wild Mallards (*Anas platyrhynchos*) Based on High-Throughput Sequencing Technology. Animals.

[31] Kanika N.H., Liaqat N., Chen H., Ke J., Lu G., Wang J., Wang C. (2025). Fish gut microbiome and its application in aquaculture and biological conservation. Front. Microbiol..

[32] Pan H., Liu H., Liu F., Xie J., Zhou Y., Zheng Q., Guo M. (2025). Gut microbiota: A new frontier in understanding and protecting endangered plateau schizothorax fish. Front. Microbiol..

[33] Nolet B., Bevan R., Klaassen M., Langevoord O., Van Der Heijden Y.G.J.T. (2002). Habitat switching by Bewick’s swans: Maximization of average long-term energy gain?. J. Anim. Ecol..

[34] Ahmed N.A., Gulhan T. (2022). Campylobacter in Wild Birds: Is It an Animal and Public Health Concern?. Front. Microbiol..

[35] Wu S., Jia R., Wang Y., Li J., Li Y., Wang L., Wang Y., Liu C., Jia E.M., Wang Y. (2024). Prevalence, Diversity, and Virulence of Campylobacter Carried by Migratory Birds at Four Major Habitats in China. Pathogens.

[36] Silva J., Leite D., Fernandes M., Mena C., Gibbs P.A., Teixeira P. (2011). *Campylobacter* spp. as a Foodborne Pathogen: A Review. Front. Microbiol..

[37] Kaakoush N.O., Castaño-Rodríguez N., Mitchell H.M., Man S.M. (2015). Global Epidemiology of Campylobacter Infection. Clin. Microbiol. Rev..

[38] Navarro J., Grémillet D., Afán I., Miranda F., Bouten W., Forero M.G., Figuerola J. (2019). Pathogen transmission risk by opportunistic gulls moving across human landscapes. Sci. Rep..

[39] Mencía-Gutiérrez A., García-Peña F.J., González F., Pastor-Tiburón N., Pérez-Cobo I., Marín M., Martín-Maldonado B. (2024). Exploring the Prevalence and Resistance of Campylobacter in Urban Bird Populations. Vet. Sci..

[40] Mourkas E., Valdebenito J.O., Marsh H., Hitchings M.D., Cooper K.K., Parker C.T., Székely T., Johansson H., Ellström P., Pascoe B. (2024). Proximity to humans is associated with antimicrobial-resistant enteric pathogens in wild bird microbiomes. Curr. Biol..

[41] Magne F., Gotteland M., Gauthier L., Zazueta A., Pesoa S., Navarrete P., Balamurugan R. (2020). The Firmicutes/Bacteroidetes Ratio: A Relevant Marker of Gut Dysbiosis in Obese Patients?. Nutrients.

[42] Gerritsen J., Hornung B., Renckens B., van Hijum S.A.F.T., Martins Dos Santos V.A.P., Rijkers G.T., Schaap P.J., de Vos W.M., Smidt H. (2017). Genomic and functional analysis of *Romboutsia ilealis* CRIBT reveals adaptation to the small intestine. PeerJ.

[43] Wu C., Yang F., Zhong H., Hong J., Lin H., Zong M., Ren H., Zhao S., Chen Y., Shi Z. (2024). Obesity-enriched gut microbe degrades myo-inositol and promotes lipid absorption. Cell Host Microbe.

[44] Wang Y., Xu X., Chen H., Yang F., Xu B., Wang K., Liu Q., Liang G., Zhang R., Jiao X. (2023). Assessment of beneficial effects and identification of host adaptation-associated genes of *Ligilactobacillus salivarius* isolated from badgers. BMC Genom..

[45] Yang Y., Song X., Wang G., Xia Y., Xiong Z., Ai L. (2024). Understanding *Ligilactobacillus salivarius* from Probiotic Properties to Omics Technology: A Review. Foods.

[46] Mukhopadhya I., Louis P. (2025). Gut microbiota-derived short-chain fatty acids and their role in human health and disease. Nat. Rev. Microbiol..

[47] Lan Q., Liufu S., Chen B., Wang K., Chen W., Xiao L., Liu X., Yi L., Liu J., Xu X. (2025). Gut-resident *Phascolarctobacterium succinatutens* decreases fat accumulation via MYC-driven epigenetic regulation of arginine biosynthesis. npj Biofilms Microbiomes.

[48] Bakermans C. (2018). Adaptations to marine versus terrestrial low temperature environments as revealed by comparative genomic analyses of the genus *Psychrobacter*. FEMS Microbiol. Ecol..

[49] Mi J., Liu K., Ding W., Zhang M.-H., Wang X.-F., Shaukat A., Rehman M.U., Jiao X.-L., Huang S.-C. (2023). Comparative analysis of the gut microbiota of wild wintering whooper swans (*Cygnus cygnus*), captive black swans (*Cygnus atratus*), and mute swans (*Cygnus olor*) in Sanmenxia Swan National Wetland Park of China. Environ. Sci. Pollut. Res..

[50] Krawczyk B., Wityk P., Gałęcka M., Michalik M. (2021). The Many Faces of *Enterococcus* spp.—Commensal, Probiotic and Opportunistic Pathogen. Microorganisms.

[51] Giraffa G., Oliveira M. (2024). Editorial: *Enterococcus* spp. -transmission, pathogenesis, host-pathogen interaction, prevention and treatment. Front. Microbiol..

